# Developmental changes in word recognition threshold from two to five years of age in children with different middle ear status

**DOI:** 10.1080/14992020701331570

**Published:** 2007-08-06

**Authors:** Amanda J. Hall, Kevin J. Munro, Jon Heron

**Affiliations:** * Centre for Hearing and Balance Studies, University of Bristol, UK; † School of Psychological Sciences, University of Manchester, UK; § ALSPAC, University of Bristol, UK

**Keywords:** Word recognition, OME, ALSPAC

## Abstract

The aims were to: (1) provide word recognition thresholds (WRTs) at 31, 43, and 61 months of age; (2) investigate developmental changes over time; (3) investigate the relationship between OME and WRT, and (4) investigate the relationship between WRT and hearing thresholds. Around 1000 children were tested longitudinally as part of the ALSPAC study, using an adaptive measure of word recognition in quiet. Mean WRTs were 28, 23, and 23 dB (A) at 31, 43, and 61 months, respectively. Normal auditory development is associated with a mean improvement in WRT of 5 dB between age 31 and 61 months. There was a mean increase in WRT of 5 dB and 15 dB when OME was present in one and two ears, respectively. Thus, both unilateral and bilateral OME results in a detrimental effect on hearing ability for speech. Additionally, early and ‘persistent’ OME is associated with greater disability. However by 61 months, previous OME status was not significant. To our knowledge, this is the largest longitudinal study reporting WRT in preschool children with different middle ear status.

Many studies have reported developmental changes in auditory abilities including pure-tone thresholds (in quiet and noise), frequency discrimination and resolution, and temporal resolution (Schneider & Trehub, 1992). Differences also exist in speech detection, discrimination and recognition, with infants requiring a higher intensity and a more favourable signal-to-noise ratio to achieve the same level of performance as adults (Nozza, 2002). These adult-infant differences may reflect underlying differences in auditory physiology and/or task-related differences such as motivation and attention (Nozza, 1995).

This study focuses on word recognition threshold, which is known to improve from infancy, through childhood to adulthood. For example, Elliott et al (1979) compared word recognition threshold (71% correct) for monosyllabic nouns in children (ranging in age from five to ten years) and adults. They demonstrated a significant improvement in mean performance of approximately 9 dB as the children increased in age. Performance at age ten years was similar to adults. Jerger and Jerger (1982) summarized the results of several studies that examined children's word recognition thresholds (50% correct) for monosyllabic words, at three to four, and five to six years of age using different test materials. They reported an improvement of 4 to 6 dB between the two age groups. Palmer et al (1991) studied 66 children from two to thirteen years of age and reported a 0.5 dB improvement in word recognition threshold for every one-year increase in age. However, Summerfield et al (1994) used the same test material with 215 children, aged two to thirteen years, and showed no evidence of an improvement in word recognition with age.

The studies cited previously were all cross-sectional in design. There are few investigations in the literature that have examined longitudinal changes in children's word recognition thresholds. A longitudinal study would be more sensitive to individual variation in the development of word recognition abilities.

Otitis media with effusion (OME) can have a detrimental effect on hearing, but it is not clear whether OME affects the development of word recognition threshold over time. Penn et al (2004) reported evidence suggesting that a simulated conductive hearing impairment has a greater effect on speech recognition compared to adults. Therefore, this suggests that the impact of a mildly attenuated signal (similar to that associated with mild conductive hearing loss) may result in greater speech perception difficulties in infants and children than in adults.

There is a significant relationship between the word recognition threshold and the better ear pure-tone average (500, 1000, 4000 Hz: Palmer et al, 1991; Summerfield et al, 1994). Summer-field et al (1994) evaluated 215 children from a clinical population using the automated toy discrimination test, and described the relationship with better ear pure-tone average as 0.82*word recognition threshold – 9.64 dB. This relationship enables the better ear pure-tone average to be estimated from the word recognition threshold results in children unable to complete a frequency specific hearing assessment. These results have not been repeated on a larger sample of children.

Thus there are few large, longitudinal studies of word recognition threshold in preschool children with normal hearing or experiencing temporary hearing impairment associated with OME. It is also uncertain whether, or how, the hearing impairment associated with OME interacts with the development of word recognition in children. Clinical audiologists make repeated measures of word recognition in children who are being followed for recurrent OME, and data on typical children with histories of OME would be useful for planning follow-up and intervention strategies.

The aims of this study were to provide normative, longitudinal word recognition threshold data at ages 31 months (∼ 2.5 years), 43 months (∼ 3.5 years), and 61 months (∼ 5 years) of age, to investigate developmental changes over time, to investigate the relationship between OME and word recognition performance, and to investigate the relationship of word recognition threshold with hearing threshold level.

## Methods

### Subjects

This study was nested within the Avon Longitudinal Study of Parents and Children (ALSPAC). ALSPAC is a large on-going longitudinal study of parents and children (14 541 mothers), designed to investigate the effect of gene-environment interaction on all aspects of child development. Mothers were enrolled who were expected to give birth between April 1991 and December 1992; for further details of the ALSPAC study and methodology refer to Golding et al (2001). A 10% sample of the ALSPAC cohort, known as the Children in Focus (CiF) group, attended clinics at the University of Bristol at various time intervals between 4 to 61 months of age. The CiF group were chosen at random from the last 6 months of ALSPAC births (1432 families attended at least one clinic). Excluded were those mothers who had moved out of the area or were lost to follow-up, and those partaking in another study of infant development in Avon. Of those families, 1135 (79.3%), 1065 (74.4%), and 994 (69.4%) attended at 31, 43, and 61 months respectively. A little over 50% of children were boys (55%, 56%, and 55% at 31, 43, and 61 months, respectively). Comparison of those families attending the CiF clinic to the rest of the ALSPAC cohort, showed significant differences in maternal educational level (educated below ‘O’ Level standard, 24 & 30.8% for CiF and ALSPAC, respectively; p <0.001), maternal age (teenage mothers, 3.3% & 4.9% for CiF and ALSPAC, respectively; p = 0.006) and housing (local authority accommodation, 11.1% & 14.8% for CiF and ALSPAC, respectively; p <0.001). Ethical approval was obtained from the ALSPAC Ethics and Law Committee.

### Hearing measures

The study used an automated version of the McCormick toy discrimination test known as the Institute of Hearing Research (IHR)-McCormick automated toy discrimination test (ATT) (Ousey et al, 1989; Palmer et al, 1991; Summerfield et al, 1994). The ATT measures the minimum sound level at which a child can identify words presented in quiet in the sound field. This word recognition threshold provides a direct measure of the ease with which a child can identify speech and is a surrogate measure of auditory sensitivity. It was designed to be applicable to most children with a developmental age of two years and above, and is the most commonly used test of speech recognition in preschool children within UK paediatric audiology services. The test material consists of seven pairs of toys with acoustically similar names (cup/duck, tree/key, man/lamb, fork/horse, spoon/shoe, cow/house and plate/plane). It uses a digital voice recording and the child is instructed to identify specific items e.g. ‘show me the *cup*’. An adaptive algorithm is used to determine the word recognition threshold (WRT), the stimulus level which the child gives criterion performance of 71%. Note that previous literature on the ATT describes the ‘word discrimination threshold’ (WDT); however, the more appropriate term “word recognition threshold” is used in this study.

The initial presentation level is 72 dB (A), reduced by 12 dB for every correct response. When the child makes an error, the level is increased by 12 dB. Following the next correct response a two-down, one-up adaptive procedure, in 6 dB steps, is used. Testing is complete once six reversals have been made. The WRT is the mean of the stimulus level at the six reversals. The WRT can be calculated using a fewer number of reversals if the child becomes restless or inattentive. A comparison was made of the mean WRT obtained in the children who completed the six reversals versus those that completed fewer reversals.

Testing was performed in a sound field, using a speaker positioned at 0 degrees azimuth. Only toys that the child recognized were included in the test. The test took approximately five to ten minutes to complete. The WRT, number of word-pairs used, and the number of completed reversals were recorded.

Middle-ear function was assessed using a Kamplex AT2 tympanometer in accordance with the British Society of Audiology ‘Recommended procedure for tympanometry’ (British Society of Audiology, 1992). The resulting tympanograms were coded according to the Fiellau-Nikolajsen's modification of Jerger's classification (Fiellau-Nikolajsen, 1983) by an audiological scientist; Type A: peak at +100 to −100 daPa; Type C1: peak at −101 to −200 daPa; Type C2: peak at −201 to −300 daPa; Type B: flat trace, no middle-ear pressure recorded. A total of 10% of the tympanograms was assessed by an independent, experienced paediatric audiologist for quality assurance purposes. The experienced audiologist changed the tympanometric category in less than 4% of cases (see Midgley et al [2000] for further details). Children's middle-ear status was classified into bilateral normal (type A or C1 tympanograms), bilateral middle-ear effusions (type B tympanograms), unilateral middle-ear effusion (type A or C1 tympanogram in one ear, with type B in the other ear), and other (grommet, perforation, or type C2 tympanogram in at least one ear). According to Takata et al (2003), using type B tympanograms to identify OME results in a sensitivity of 81% and a specificity of 75%. Grouping type B and C2 increases sensitivity (94%) at the expense of specificity (62%). In the present study, specificity was regarded as more important than sensitivity (i.e. to minimize the number of normal ears classified as OME); therefore, type B tympanograms alone were used to identify OME cases (see later).

At 61 months of age, air-and bone-conduction hearing threshold levels (HTL) were measured using a calibrated Kamplex AD12 pure-tone audiometer. The test order was designed to obtain ear-specific, mid-and high-frequency air conduction thresholds first, and then to measure additional frequencies if the child was still cooperative. Hearing thresholds were measured in the following order: 1000 and 4000 Hz in the right ear then the left ear, 8000 Hz in the right and then the left ear. A re-check at 1000 Hz in the right ear was then measured. If the child was still cooperative (and time permitted), 500 Hz was measured in the right then the left ear, followed by 2000 Hz in the right then the left ear. Bone conduction thresholds were measured at 1000 Hz.

All testing was performed in a quiet room, with a background noise level no greater than 35 dB (A). Testing was performed by qualified audiologists and staff trained specifically for this purpose. Statistical analyses were performed using linear regression and repeated measures ANOVA (SPSS version 12.0). WRT was used as the dependent variable, and adjustments were made for gender using multivariable regression. The WRT was also analysed with respect to middle-ear status.

## Results

### Test considerations

**Figure 1.**
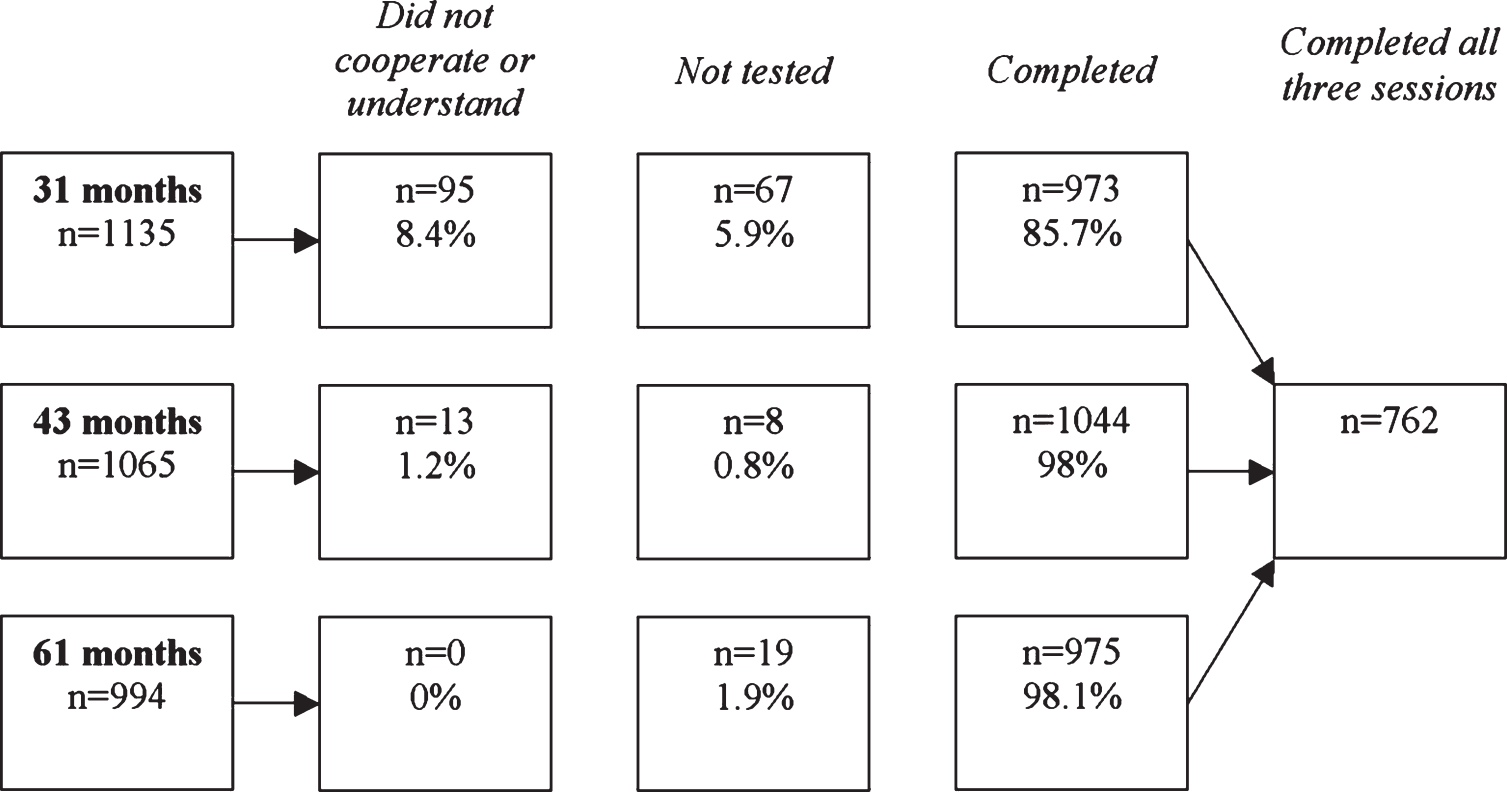
Summary of numbers of children completing the test at each session, the numbers of children who would not cooperate or did not understand the test, and those for which testing was not done (e.g. due to time constraints).

The number of children completing the test at each age is shown in Figure 1. Longitudinal analyses were restricted to the 762 cases where data were available at each time point. Cross-sectional analyses used all cases available at 31, 43, and 61 months (973, 1044, and 975, respectively). The percentage of children who could not cooperate for testing decreased from 8.4% at 31 months to 1.2% and less from 43 months of age onwards. Lack of time was the main reason for some children not being tested.

At 31 months of age, 57% of children recognized all toy-pairs: by 61 months of age, this has increased to 99%. There was no significant difference in mean WRT according to the number of toy pairs used (F [3, 967] =0.05; p=0.99). The number of children completing the maximum number of reversals increased from 94% at 31 months to more than 99% in the older age groups. At 31 months there was a significant difference in WRT according to the number of reversals used (F [1, 970] 11.89; p=0.001), with fewer reversals associated with a higher WRT (see Table 1). The number of children completing six reversals who had bilateral normal middle ears, bilateral middle-ear effusion, unilateral middle-ear effusion, and other middle-ear status is 618 (71.4%), 91 (10.5%), 90 (10.4%), and 66 (7.6%), respectively. The corresponding numbers for children who could not complete six reversals are 39 (70.9%), 5 (9.1%), 7 (12.7%), and 4 (7.3%), respectively. Tympanometry data were missing from 52 cases. There was no significant difference in middle-ear status between the six reversals and fewer than six reversals groups (χ^2^ (3, N = 920) = 0.37, p = 0.945).

**Table 1.** Mean WRT at 31 months, by number of toy pairs and number of reversals

*Number of toy pairs*	Mean WRT*	*n*	*%*
3	29	1	0.1
4	31.1 (15.6)	8	0.8
5	31.7 (10.0)	71	7.3
6	31.6 (8.7)	338	35
7	31.8 (7.4)	554	57
Total	31.7 (8.1)	972	100
*Number of reversals*			

<6	35.3 (12.0)	58	6
6	31.5 (7.8)	914	94
Total	31.7 (8.1)	972	100

* Word recognition threshold dB (A), standard deviation in brackets

Note: Number of cases is 972 rather than 973, as there was one subject with a valid WRT result but missing data for number of toy pairs and number of reversals, so they were not included in these analyses

### WRT and the influence of age

The mean WRT for children with normal middle-ear function was examined. A total of 340 children with data available at all time points were included in the analysis. Mean WRT improved by 5 dB as age increased from 31 to 61 months (see Table 2). This difference was statistically significant on a repeated-measures ANOVA (F [1.9, 648]=207.1; p≤0.001). The data were then analysed using paired *t*-tests. The differences between 31 and 43 months (mean difference = 5.4 dB (95% CI: 4.8, 6.0 dB), and 31 and 61 months (mean difference 5.3 dB (95% CI: 4.6, 5.9 dB) were statistically significant. The difference between 43 and 61 months was not significant (95% CI: −0.6, 0.4 dB).

**Table 2.** Mean WRT in cases with normal middle ear function at 31, 43, and 61 months of age. Only subjects with complete data at all three time points were included (n =340).

*Age (months)*	*31*	*43*	*61*
WRT*	28.5 (5.0)	23.1 (3.6)	23.2 (3.7)

* Word recognition threshold dB (A), standard deviation in brackets

### WRT and the influence of OME

The mean WRT for children with different middle-ear status was examined at each age using multivariable regression, where WRT was the dependent variable and middle-ear status the independent variable. As boys may be more prone to OME (Teele et al, 1989), and there may be a gender difference in hearing thresholds (Pearson et al, 1995), gender was controlled for in the regression model. In this instance, the effect of gender was negligible (values changed by B5%) and Table 3 shows the adjusted values only. The best WRT was obtained in the children with no evidence of OME. There was a statistically significant effect of OME compared to normal middle-ear function at 31 months, (F [3, 916] 201.6, p ≤0.001); 43 months, (F [3, 1021] 288.7, p ≤0.001); and 61 months (F [3, 945] 236.5, p 50.001). Compared to the normal children, the mean WRT of children with unilateral and bilateral OME was elevated by 5 dB and 15 dB, respectively.

**Table 3.** Association between mean WRT and middle ear status at 31, 43, and 61 months of age.

	*Age*								
	
	*31 months*			*43 months*			*61 months*		
			
Middle-ear status*	*Mean WRT*†	*n*	*Effect size (dB)*‡	*Mean WRT*†	*n*	*Effect size (dB)*‡	*Mean WRT*†	*n*	*Effect size (dB)*‡
Bilateral normal	29.2 (6.0)	657	Reference	23.7 (4.2)	665	Reference	23.5 (4.0)	703	Reference
Unilateral OME	34.2 (6.0)	98	5.1 (3.7–6.4)	28.1 (4.8)	110	4.4 (3.3–5.5)	28.8 (4.3)	59	5.3 (4.1–6.6)
Bilateral OME	46.0 (8.7)	96	16.8 (15.4–18.1)	39.2 (10.1)	117	15.5 (14.5–16.6)	38.5 (8.9)	68	15.0 (13.8–16.2)
Other	32.7 (5.9)	70	3.4 (1.9–5.0)	28.5 (5.2)	134	4.8 (3.8–5.8)	28.0 (5.5)	120	4.5 (3.6–5.4)
			**p≤0.001**			**p≤0.001**			**p≤0.001**

* Middle-ear status: bilateral normal-type A or C1 tympanograms; bilateral middle-ear effusions-type B tympanograms; unilateral middle-ear effusion-type A or C1 tympanograms in one ear with type B in the other ear; other-grommet, perforation, or type C2 tympanogram in at least one ear

† Mean word recognition threshold dB (A), adjusted for gender, standard deviation in brackets

‡ Estimated differences in WRT with reference to bilateral normal middle-ear function at each age, 95% confidence interval shown in brackets

### Changes in WRT and OME status

The relationship between WRT and current (or previous) bilateral OME status was investigated; i.e. WRT at 43 months and OME status at 43 or/and 31 months, and WRT at 61 months and OME status at 61 and/or 43 months. Multivariable regression analysis was used with WRT as the dependent variable, and change in OME status as the independent variable. The change in OME status was categorized according to whether bilateral OME was: (1) present at both time points, (2) present at the first time point but not the second, (3) present at the second time point but not the first, or (4) absent at both time points. The data were controlled for gender in the regression model. The effect of gender was negligible (values changed by <5%) and Table 4 shows the adjusted values only. The 43-month WRT was 8 dB poorer in children with bilateral flat tympanograms measured 12 months earlier. The 61-month WRT was 5 dB poorer in children with bilateral flat tympanograms recorded 18 months earlier. The longitudinal data show that children with bilateral flat tympanograms at 43 months have an elevated WRT and this is related to middle-ear status 12 months earlier: the mean elevation in WRT was 13 dB and 19 dB in children with normal and flat tympanograms at 31 months, respectively (i.e. persistence of OME from an early age results in a poorer WRT). Children with bilateral OME at 61 months have a mean elevation in WRT of 14–15 dB and this is independent of OME status at 43 months.

**Table 4.** Association between WRT and current (and previous) OME status.

OME*		*Mean WRT*†		OME*				*Mean WRT*†		*WRT significantly different (p≤0.001) to groups*
							
*31 m*	*N*	*43 m*	*Effect size (dB)*‡		*31 m*	*43 m*	*N*	*43 m*	*Effect size (dB)*‡	
−	509	24.8 (24.2, 25.4)	Reference	1	−	−	392	23.3 (22.8, 23.8)	Reference	3, 4
+	78	32.8 (31.4, 34.3)	8.0 (6.5, 9.6)	2	+	−	20	23.3 (21.1, 25.6)	0.1 (−2.3, 2.4)	3, 4
			**p≤0.001**	3	−	+	28	36.5 (34.6, 38.4)	13.3 (11.3, 15.2)	1, 2, 4
				4	+	+	31	42.3 (40.5, 44.1)	19.0 (17.2, 20.9)	1, 2, 3
									**p≤0.001**	
*43 m*		*61 m*			*43 m*	*61 m*		*61 m*		

−	497	24.2 (23.7, 24.7)	Reference	1	−	−	424	23.2 (22.8, 23.6)	Reference	3, 4
+	84	29.2 (28.0, 30.4)	5.1 (3.8, 6.4)	2	+	−	28	24.0 (22.4, 25.7)	0.9 (−0.9, 2.6)	3, 4
			**p≤0.001**	3	−	+	16	38.9 (36.7, 41.1)	15.8 (13.5, 18.0)	1, 2
				4	+	+	21	37.3 (35.4, 39.3)	14.2 (12.2, 16.1)	1, 2
									**p≤0.001**	

* Middle ear status: +OME present bilaterally (bilateral type B tympanograms); − OME absent bilaterally (bilateral type A or C1 tympanograms)

† Mean word recognition threshold dB (A), adjusted for gender, 95% confidence interval shown in brackets

‡ Estimated differences in WRT (dB) with reference to bilateral normal middle-ear function, 95% confidence interval shown in brackets

### WRT and hearing threshold level

**Figure 2.**
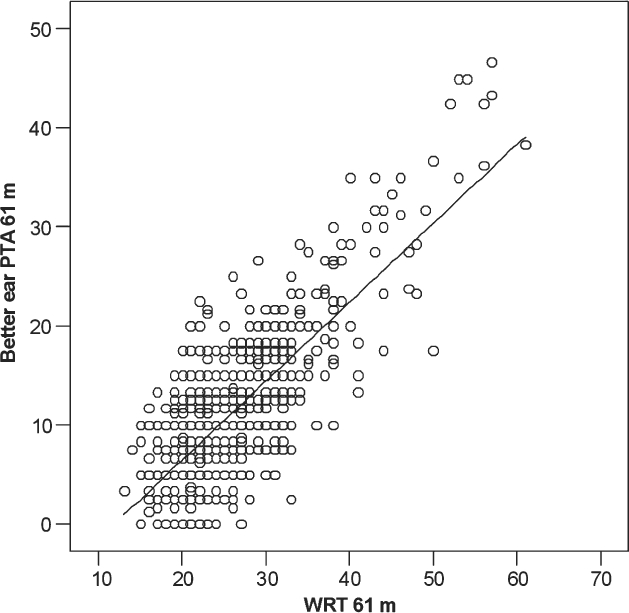
Relationship between 61 month WRT and the better ear PTA (500, 1000, 2000, and 4000 Hz). Linear regression line plotted.

Linear regression was used to evaluate the relationship between WRT and the better ear pure-tone average (PTA) thresholds for various frequency combinations. The results are summarized in Table 5 and the relationship between WRT and the better ear PTA (500, 1000, 2000, and 4000 Hz) is shown in Figure 2. The correlation between WRT and PTA was similar across the different frequency combinations.

**Table 5.** Linear regression analysis relating 61 month WRT (independent variable) to the PTA (dependent variable). N=962.

PTA frequencies (Hz)*	*R*†	*Constant*	*Slope*‡
500, 1000, 2000, 4000	0.7	−9.47	0.80 (0.75, 0.84)
500, 1000, 4000	0.7	−9.56	0.80 (0.76, 0.85)
1000, 4000	0.7	−10.57	0.82 (0.77, 0.87)

* PTA: pure-tone average

† R: correlation coefficient

‡ 95% confidence interval shown in brackets

## Discussion

The ATT was successfully performed in over 85% of children at 31 months, and in excess of 98% when 43 months or older. These figures are similar to those reported by Summerfield et al (1994), although the present study was based on a much larger sample of preschool children where any difficulties are likely to be more common. The number of toy pairs did not significantly affect the WRT measured, and shows that fewer toy pairs can be used in clinical practice if necessary. This is an important finding for clinicians. The WRT was 4 dB higher in children who were unable to complete the full number of reversals. A child with a short attention span may respond inconsistently and only at supra-threshold levels. This would explain why the test was terminated before all the desired reversals in the adaptive sequence were completed and why the WRT is elevated. Clinicians should be aware that terminating the test early may result in a higher WRT and this weakens the relationship with pure-tone thresholds.

For children with normal middle-ear function, there was a mean improvement in WRT of 5 dB between 31 and 43 months, but no further improvement at 61 months. These are novel, longitudinal results that show an improvement in speech recognition with age. The improvement is unlikely to be related to practice effects as the interval between test sessions for each child was 12 months. The 5 dB improvement in WRT from age 2½ to age 5 years was obtained from a sample of 340 children: to our knowledge, this represents one of the largest studies of speech recognition in preschool children. These data are in agreement with Palmer et al (1991). The improvements are larger in the present study but the reason for this is unclear.

The mean WRT in normal children at 5 years of age was 23 dB (A). This equates to a pure-tone hearing threshold in the better ear (at 500, 1000, and 4000 Hz) of 8.8 dB HL (95% CI, 7.9–10.0 dB HL). This was obtained from a sample of 703 children; again, this represents one of the largest normative studies published to date. Previous work on eight normally hearing adults by Ousey et al (1989) revealed a mean WRT of 18.6 dB (A). This suggests that the WRT of 5-year old children will improve by a further 5 dB as they reach adulthood. Thus, the mean improvement in word recognition from 2½ years to adulthood is likely to be of the magnitude of 11 dB.

Summerfield et al (1994) reported a mean test-retest difference of 0.2 dB in their clinical population of 202 children (age range 2 to 13.4 years). However, the standard deviation of the difference was 3.5 dB, giving a 95% confidence interval of +/−7dB. Therefore the developmental changes reported in the present study are small, and any changes over time within one child may be masked by the test-retest reliability.

There was a significant effect of OME on WRT at each time point. Irrespective of age, the mean difference in WRT between children with bilateral OME and normal middle-ear function was 15 dB. This mean difference is greater than that associated with normal developmental changes and represents an increase of better ear hearing thresholds of +12 dB (when using the regression formula in Table 5). Bilateral OME had the greatest effect at 31 months, partly as a result of the developmentally poorer thresholds at this age, but also because the range of thresholds at 31 months was greater than at the other ages tested. Sabo et al (2003) reported similar results with regard to hearing threshold and OME. They found a mean difference of 10 to 15 dB in threshold between children with normal middle-ear function and bilateral OME. The detrimental effect on mean WRT is a significant finding because it demonstrates that OME causes a disability, and this has relevance and importance to the counselling for parents of children with OME.

Comparison of the mean WRT in children with unilateral OME versus normal middle-ear function showed an elevated mean threshold of approximately 4 to 5 dB. This difference in threshold is likely to arise from an absence of binaural summation. At threshold, the binaural versus monaural advantage is approximately +3 dB for a variety of stimuli including speech (Shaw et al, 1947; Reynolds & Stevens, 1960). These data show that unilateral OME can have a detrimental effect on word recognition threshold, and suggests that it may be equally important to counsel parents of children with unilateral as well as bilateral OME on the importance of good listening conditions and hearing tactics.

The OME groups in this study were classified according to tympanometric status, and the classification process used may have affected the WRT for the different groups. Given that no test is 100% sensitive and 100% specific, the OME group will include some normals, and the normal group will include some OME cases. As a result, the mean WRT of the OME group may have been underestimated by the inclusion of normal cases. The larger numbers in the normal group mean that the inclusion of some OME cases is unlikely to have had much effect on the mean WRT.

Previous and current OME status had a significant effect on WRT. Subjects with OME at 31 and 43 months had raised thresholds compared to those with OME at 43 months alone. The finding that both previous and current OME status had a significant effect on WRT between 31 and 43 months of age is an important finding and is clinically significant, i.e. early and 'persistent' OME is associated with greater disability as demonstrated by higher WRTs measured in the quiet. By 61 months, previous OME status was not important. However, this analysis only takes account of OME measured on two occasions separated by 12 months, and does not attempt to measure true persistence of OME. OME before 31 months is not accounted for, and the results may be different if a more complete history of OME persistence is used.

The relationship between WRT and hearing thresholds showed that word recognition is strongly related to hearing threshold. This relationship is remarkably similar to the one reported by Summerfield et al (1994). For example, a WRT of 30 dB (A) equates to pure-tone thresholds in the better hearing ear (at 500, 1000 and 4000 Hz) of 15 dB HL using the Summerfield data, and 14.5 dB HL using the data from the present study. The residual variability in WRT not explained by hearing thresholds is probably related to task-related variables, such as attention and behaviour. This will be explored in a companion paper.

The children from whom these results were derived showed some bias to the higher socio-economic groups. Developmental changes in WRT may actually be greater (and have a longer time course) than those shown here.

## Conclusions

The ATT can be successfully performed in most children. The number of toy pairs used does not significantly affect WRT; however, terminating the test before six reversals gives a significantly increased WRT. There is an improvement of 5 dB in mean WRT between age 31 and 61 months. This finding is an important contribution to our knowledge concerning normal auditory development. Unilateral and bilateral OME results in a mean increase in WRT of 4–5 dB and 15 dB, respectively. Therefore, both unilateral and bilateral OME result in a detrimental effect on hearing ability of speech in quiet. For example, unilateral OME at 61 months is equivalent to performance with normal middle-ear function at 31 months. In addition, early and ‘persistent’ OME is associated with greater disability. However, there were no apparent long-term side effects, in terms of word recognition in quiet, if tympanometry was normal by age 61 months. These findings are important and clinically relevant when counselling the families of children who have OME. To our knowledge, this is the largest study providing longitudinal data on WRT for different ages and with different middle-ear status.

## Abbreviations

OME: Otitis media with effusion
WRT: Word recognition threshold
ALSPAC: Avon Longitudinal Study for Parents and Children
